# Effectiveness of the capsaicin 8% patch in the management of peripheral neuropathic pain in European clinical practice: the ASCEND study

**DOI:** 10.1186/s12883-017-0836-z

**Published:** 2017-04-21

**Authors:** Colette Mankowski, Chris D. Poole, Etienne Ernault, Roger Thomas, Ellen Berni, Craig J. Currie, Cecil Treadwell, José I. Calvo, Christina Plastira, Eirini Zafeiropoulou, Isaac Odeyemi

**Affiliations:** 1 Astellas Pharma Europe Ltd, 2000 Hillswood Drive, Chertsey, KT16 0PS UK; 2 Astellas Pharma Europe B.V., Leiden, The Netherlands; 3 Pharmatelligence, Cardiff, UK; 4 Cardiff University, Cardiff, UK; 5 Complejo Hospitalario de Navarra, Pamplona, Spain; 6 Evangelismos General Hospital, Athens, Greece

**Keywords:** Capsaicin 8% patch, Neuropathy, Pain management, Peripheral neuropathic pain, Topical analgesic, Numeric pain rating scale, Health-related quality of life

## Abstract

**Background:**

In randomised studies, the capsaicin 8% patch has demonstrated effective pain relief in patients with peripheral neuropathic pain (PNP) arising from different aetiologies.

**Methods:**

ASCEND was an open-label, non-interventional study of patients with non-diabetes-related PNP who received capsaicin 8% patch treatment, according to usual clinical practice, and were followed for ≤52 weeks. Co-primary endpoints were percentage change in the mean numeric pain rating scale (NPRS) ‘average daily pain’ score from baseline to the average of Weeks 2 and 8 following first treatment; and median time from first to second treatment. The primary analysis was intended to assess analgesic equivalence between post-herpetic neuralgia (PHN) and other PNP aetiologies. Health-related quality of life (HRQoL, using EQ-5D), Patient Global Impression of Change (PGIC) and tolerability were also assessed.

**Results:**

Following first application, patients experienced a 26.6% (95% CI: 23.6, 29.62; *n =* 412) reduction in mean NPRS score from baseline to Weeks 2 and 8. Equivalence was demonstrated between PHN and the neuropathic back pain, post-operative and post-traumatic neuropathic pain and ‘other’ PNP aetiology subgroups. The median time from first to second treatment was 191 days (95% CI: 147, 235; *n =* 181). Forty-four percent of all patients were responders (≥30% reduction in NPRS score from baseline to Weeks 2 and 8) following first treatment, and 86.9% (*n =* 159/183) remained so at Week 12. A sustained pain response was observed until Week 52, with a 37.0% (95% CI: 31.3, 42.7; *n =* 176) reduction in mean NPRS score from baseline. Patients with the shortest duration of pain (0–0.72 years) experienced the highest pain response from baseline to Weeks 2 and 8. Mean EQ-5D index score improved by 0.199 utils (responders: 0.292 utils) from baseline to Week 2 and was maintained until Week 52. Most patients reported improvements in PGIC at Week 2 and at all follow-up assessments regardless of number of treatments received. Adverse events were primarily mild or moderate reversible application site reactions.

**Conclusion:**

In European clinical practice, the capsaicin 8% patch provided effective and sustained pain relief, substantially improved HRQoL, improved overall health status and was generally well tolerated in a heterogeneous PNP population.

**Trial registration:**

NCT01737294 Date of registration - October 22, 2012.

## Background

Peripheral neuropathic pain (PNP) is caused by a lesion or disease involving the somatosensory system [1]. Common causes of PNP include traumatic nerve injury, surgery, diabetes, herpes zoster infection, cancer, chemotherapy and human immunodeficiency virus (HIV) infection [2]. PNP affects 7–8% of the population in Europe [3, 4] and can negatively impact quality of life, psychological wellbeing, sleep and work productivity [5].

The latest treatment guidance from the Neuropathic Pain Special Interest Group (NeuPSIG) of the International Association for the Study of Pain recommends several options for first line treatment of neuropathic pain (NP), including calcium α_2_-δ ligands (e.g. pregabalin, gabapentin), serotonin/norepinephrine reuptake inhibitors and tricyclic antidepressants [6]. Despite proven efficacy, these therapies have limitations including inadequate pain relief, lengthy dose-titration periods, multiple daily dosing, dose-limiting adverse events, suboptimal adherence due to adverse events, and the potential for abuse and addiction [7–9].

NeuPSIG guidance suggests tramadol, the lidocaine 5% patch and the 179 mg (8% w/w) capsaicin patch as second line treatment options for patients with neuropathic pain [6]. Capsaicin is a selective, potent and high-affinity agonist for the transient receptor potential vanilloid type 1 (TRPV1) ion channel complex [10]. Application of high-dose capsaicin at the site of pain can defunctionalise TRPV1 leading to disruption of mitochondrial respiration and retraction of the nerve fibres, thereby reducing the pain response [10]. Localised treatment with the capsaicin 8% patch limits the potential for drug–drug interactions and avoids the need for dose adjustment in the elderly or in patients with renal or hepatic impairment [11].

The capsaicin 8% patch has been shown to reduce pain compared with placebo for patients with post-herpetic neuralgia (PHN), HIV-associated neuropathy (HIV-AN), and painful diabetic peripheral neuropathy [12–17]. In non-diabetic patients with a variety of PNP aetiologies, the capsaicin 8% patch demonstrated non-inferior pain relief versus pregabalin, with a more rapid onset of pain relief and fewer systemic side effects [18]. The capsaicin 8% patch is generally well tolerated with treatment-related side effects mostly limited to application site reactions such as erythema [12, 14].

A 12-week, non-interventional study of a single capsaicin 8% patch treatment demonstrated effectiveness and suggested a benefit of early treatment within six months of diagnosis [19]. The aim of this non-interventional study was long-term monitoring (52 weeks) of non-diabetic patients with PNP undergoing treatment with the capsaicin 8% patch in a real-world setting. This work reports the efficacy, re-treatment pattern, tolerability and health-related quality of life (HRQoL) associated with capsaicin 8% patch treatment in Europe.

## Methods

### Study design and participants

The ASCEND study (NCT01737294) was a Phase 4, multi-centre, open-label, non-interventional study (NIS) conducted between February 2012 and August 2014 in accordance with the principles of the Declaration of Helsinki, International Conference on Harmonisation Guidelines, and local ethical and legal requirements.

Patients were eligible for inclusion if they were at least 18 years old, recommended capsaicin 8% patch treatment by their treating physician, diagnosed with non-diabetic PNP and had provided written informed consent for participation in the study. Patients were excluded for the following reasons: neuropathic painful areas located only on the face, above the hairline of the scalp and/or in proximity to mucous membranes; history of diabetes mellitus; diagnosis of any major psychiatric disorder, or evidence of cognitive impairment; prior treatment with capsaicin 8% patch; hypersensitivity to capsaicin, capsaicin 8% patch excipients/adhesives, and/or local anaesthetics; participation in any other clinical study and/or receipt of an investigational drug within 30 days prior to screening visit; history of substance abuse (including alcoholism).

A detailed medical history was taken with particular emphasis on the primary PNP diagnosis. Patients were classified into one of six aetiology groups: PHN; HIV-AN; neuropathic back pain (NBP), including cases secondary to radiculopathy, polyneuropathy, plexopathy and ankylosing spondylitis; cancer-related neuropathic pain (CRNP); post-operative and post-traumatic neuropathic pain (PONP); ‘other’ neuropathies. Prior and concomitant medications were recorded at baseline and patients were categorised by the treating physician as being in the primary (first treatment received for NP), secondary (second treatment received for NP) or tertiary (at least third treatment received for NP) stage of the treatment pathway. The duration of pre-existing PNP was recorded.

### Treatment

**Fig. 1:**
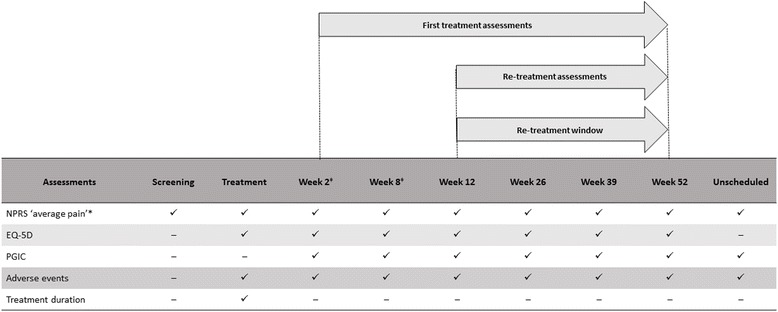
Schedule of patient assessments. *Numeric pain rating scale (NPRS) ‘average pain during the last seven days’ score was recorded at the screening visit and used as the baseline pain score. NPRS ‘average pain during the last 24 h’ score was recorded at treatment visits and assessments; ^‡^Week 2 and Week 8 assessments were performed only after first capsaicin 8% patch treatment. EQ-5D, EuroQol 5 Dimension 3-level; PGIC, patient global impression of change

Study medication was prescribed in routine clinical practice. At each treatment visit, the size and location of the patient’s painful area was assessed to determine the required area of treatment. Each capsaicin 8% patch contained 179 mg of capsaicin (640 μg per 1 cm^2^) and up to four patches were allowed per treatment. Multiple treatment areas were possible; the recommended treatment time was 30 min for the foot and 60 min for other anatomical sites. Patients were followed up by phone or during clinic visits (Fig. 1). Scheduled follow-up contact at Week 2 and Week 8 was made after first treatment only. Subsequent follow-up contact was made at Week 12, Week 26, Week 39 and Week 52. Multiple treatments with the capsaicin 8% patch were allowed, although intervals of at least 90 days between each application were recommended, consistent with the summary of product characteristics [20].

### Efficacy and tolerability assessments

Patients assessed the intensity of their pain using an 11-point numeric pain rating scale (NPRS) ranging from 0 (no pain) to 10 (worst imaginable pain) [21]. NPRS ‘average pain during the last seven days’ score was recorded at the screening visit and used as baseline pain in all related analyses. NPRS ‘average pain during the last 24 h’ score was recorded at each treatment visit/assessment (prior to patch application). Sensitivity analysis was performed to exclude the effect of treatment outside of the recommended application time (<90% and ≥110%) on the pain response. HRQoL was assessed using the EuroQol 5 Dimension 3-level (EQ-5D) questionnaire [22]. The default York MVH A1 value set [23] was used to derive the EQ-5D index for all observations in this study. The change in patients’ general state of health was assessed by the patient global impression of change (PGIC) instrument [24] using a 7-point Likert scale, ranging from 1 (very much improved) to 7 (very much worse). Patients were asked to indicate how they felt ‘now’, compared with how they felt before receiving their most recent capsaicin 8% patch treatment.

The primary endpoints were: (i) the percentage change in mean NPRS ‘average pain’ score from baseline to the average of Weeks 2 and 8 following first capsaicin 8% patch treatment; and (ii) the median time between the first and second capsaicin 8% patch treatments. Secondary endpoints were: the percentage change in mean NPRS score from baseline to the average of Weeks 2 and 12 following re-treatment(s); the proportion of patients with a ≥30% or ≥50% reduction in mean NPRS score from baseline to the average of Weeks 2 and 8 for first treatment or the average of Weeks 2 and 12 for re-treatment (defined as ≥30% responders and ≥50% responders, respectively); the percentage change in mean NPRS scores from baseline to each assessment; median time between second and third treatment; number of capsaicin 8% patches used at each treatment; treatment area size at each treatment; the proportion of patients completing ≥90% of the recommended treatment duration at each application (≥27 min for the feet or ≥54 min for all other anatomical sites); change in EQ-5D index from baseline to each assessment; proportion of patients with improved overall health status versus baseline according to the PGIC (i.e. very much improved, much improved or slightly improved) at assessments; proportion of patients reporting adverse events (AEs) and serious AEs (SAEs). All study assessments were performed at scheduled follow-up visits and, with the exception of EQ-5D, at additional, unscheduled visits.

### Statistical analyses

All analyses were performed using the full analysis set (FAS), consisting of all patients who received treatment with the capsaicin 8% patch. The planned primary analysis was to test equivalence between the PHN group and each of the other PNP aetiology groups for each co-primary endpoint. The margin of equivalence for percentage change in mean NPRS score was set at ±16% (comparable to a one-point change on the NPRS scale [25]) while for time to re-treatment, it was set at ±1 month according to clinical judgement. The decision to perform inferential equivalence testing between the patients in the PHN aetiology group and those in any of the other PNP aetiology groups for the analysis of the co-primary endpoints was based on the number of PHN patients recruited and the minimum number of patients in any one of the other aetiologies required to achieve 80% power.

Based on the actual number of PHN patients (*n =* 89), for the first co-primary endpoint (percentage change in mean NPRS score from baseline), recruitment in three of the aetiology groups (NBP, PONP and ‘other’) was sufficient to achieve 80% power. However, recruitment to the CRNP and HIV-AN aetiology groups fell below the minimum threshold; therefore comparison of neither group versus PHN was performed for this co-primary endpoint. An analysis of covariance was used to adjust for gender, country and baseline ‘average pain’ as fixed-effects covariates. Least squares mean estimates were provided with 95% confidence intervals (CIs). For the second co-primary endpoint (time to re-treatment), recruitment in all PNP aetiology groups fell below the minimum threshold to achieve sufficient power for testing equivalence. Therefore only descriptive time to event statistics derived using the Kaplan-Meier method were provided for each PNP aetiology group. Missing values were presented without imputation for all analyses. A sensitivity analysis was conducted in order to demonstrate consistency with the primary endpoint. Patients treated outside of the recommended application time (˂90% [˂27 min for the feet; <54 min for all other anatomical sites] and ≥110% [≥33 min for the feet; ≥66 min for all other anatomical sites]) were excluded from this analysis.

## Results

### Patient characteristics

A total of 429 patents were enrolled in the study and 420 patients received at least one treatment with the capsaicin 8% patch (FAS). Patients were from seven European countries: Austria (7 sites, *n =* 65), Greece (11 sites, *n =* 88), Italy (8 sites, *n =* 30), Portugal (11 sites, *n =* 98), Spain (7 sites, *n =* 68), Switzerland (2 sites, *n =* 22) and United Kingdom (5 sites, *n =* 49). The median age of the study population was 61 (range 21–98) years; 39.8% of patients were male; and the most common diagnoses were PONP (47.1%, *n =* 198) and PHN (21.1%, *n =* 89) (Table 1). The proportion of patients in the primary, secondary or tertiary stage of the treatment pathway was 19.3%, 42.9% and 37.9%, respectively. Overall, patients had a median follow-up time of 370 days (interquartile range: 360–434).

**Table 1:** Patient demographics and baseline characteristics (FAS)

	PHN (*n =* 89)	HIV-AN (*n =* 5)	NBP (*n =* 50)	CRNP (*n =* 22)	PONP (*n =* 198)	Other (*n =* 56)	Overall (*n =* 420)
Gender, *n* (%)							
Male	36 (40.4)	5 (100)	17 (34.0)	5 (22.7)	75 (37.9)	29 (51.8)	167 (39.8)
Female	53 (59.6)	0	33 (66.0)	17 (77.3)	123 (62.1)	27 (48.2)	253 (60.2)
Ethnicity, *n* (%)							
Caucasian	88 (98.9)	4 (80.0)	49 (98.0)	19 (86.3)	185 (93.4)	55 (98.2)	400 (95.2)
Asian	0	1 (20.0)	0	0	2 (1.0)	0	3 (0.7)
Black/African/Caribbean	1 (1.1)	0	1 (2.0)	1 (4.5)	8 (4.0)	1 (1.8)	12 (2.9)
Mixed/multiple ethnic groups	0	0	0	1 (4.5)	1 (0.5)	0	2 (0.5)
Not recorded	0	0	0	1 (4.5)	2 (1.0)	0	3 (0.7)
Median age, years (min–max)	72 (37–98)	52 (33–59)	63 (27–90)	59 (42–76)	54 (21–83)	60 (24–86)	61 (21–98)
Median duration of pain, years (min–max)	1.0 (0.1–73.2)	0.3 (0.2–13.6)	2.6 (0–40.0)	2.2 (0.2–8.1)	2.6 (0.1–50.1)	2.2 (0.1–29.2)	2.1 (0–73.2)
Treatment pathway, *n* (%)							
Primary	13 (14.6)	1 (20.0)	20 (40.0)	2 (9.1)	35 (17.7)	10 (17.9)	81 (19.3)
Secondary	48 (53.9)	3 (60.0)	14 (28.0)	13 (59.1)	78 (39.4)	24 (42.9)	180 (42.9)
Tertiary	28 (31.5)	1 (20.0)	16 (32.0)	7 (31.8)	85 (42.9)	22 (39.3)	159 (37.9)
Baseline ‘average pain’,^a^ *n* (SD)	7.1 (2.0)	6.4 (1.5)	7.3 (2.0)	7.2 (1.5)	6.8 (1.8)	6.9 (1.8)	6.9 (1.8)
Mean number of concomitant medications,^b^ *n* (SD)	1.8 (1.5)	1.4 (1.5)	1.5 (1.6)	2.2 (1.4)	1.9 (1.5)	1.7 (1.4)	1.8 (1.5)

^a^Average pain during the 7 days prior to screening visit; ^b^Number of concomitant medications for neuropathic pain at screening visit. *CRNP*, cancer-related neuropathic pain; *FAS*, full analysis set; *HIV-AN*, HIV associated neuropathy; *NBP*, neuropathic back pain; *NPRS*, numeric pain rating scale; Other, other non-diabetic PNP; *PHN*, postherpetic neuralgia; *PONP*, post-operative and post-traumatic neuropathic pain; *SD*, standard deviation

### Treatment exposure

At first treatment, the mean number of patches used was 1.5 (standard deviation [SD] ±0.7) and the mean treatment area was 306.4 (SD ±228.2) cm^2^; both values remained consistent through successive applications (Table 2). A total of 239 (56.9%) patients received only one treatment with the capsaicin 8% patch, 181 (43.1%) patients received at least two treatments and 70 (16.7%) patients received at least three treatments. At first treatment, patches were applied to the following body areas: legs (36.2%, *n =* 152); torso (35.0%, *n =* 147); feet (16.2%, *n =* 68); hands (8.6%, *n =* 36); arms (8.3%, *n =* 35); and the head and neck (5.5%, *n =* 23), excluding areas above the hairline of the scalp and/or in proximity to mucous membranes. During first treatment, 388 patients (92.4%) completed ≥90% of the recommended duration of patch application. Similarly, 157 (94.0%) and 63 (98.4%) patients completed ≥90% of the recommended duration of patch application at second and third treatment, respectively.

**Table 2:** Treatment exposure (FAS)

	PHN (*n =* 89)	HIV-AN (*n =* 5)	NBP (*n =* 50)	CRNP (*n =* 22)	PONP (*n =* 198)	Other (*n =* 56)	Overall (*n =* 420)
Patients treated at each application, *n* (%)							
First treatment	89 (21.2)	5 (1.2)	50 (11.9)	22 (5.2)	198 (47.1)	56 (13.3)	420 (100)
Second treatment	40 (9.5)	2 (0.5)	15 (3.6)	8 (1.9)	97 (23.1)	19 (4.5)	181 (43.1)
Third treatment	16 (3.8)	–	4 (1.0)	4 (1.0)	38 (9.0)	8 (1.9)	70 (16.7)
Mean size of treatment area, cm^2^ (SD)							
First treatment	298.6 (168.1)	380.0 (138.6)	297.7 (196.9)	519.5 (404.9)	271.5 (209.7)	362.0 (263.9)	306.4 (228.2)
Second treatment	286.8 (169.3)	210.0 (99.0)	291.3 (160.6)	566.6 (387.9)	278.6 (206.4)	391.5 (300.5)	306.4 (225.0)
Third treatment	270.0 (190.6)	–	357.5 (230.7)	467.5 (113.5)	229.7 (211.8)	487.4 (412.4)	294.8 (248.8)
Mean number of patches (SD)							
First treatment	1.4 (0.6)	1.4 (0.6)	1.3 (0.6)	2.1 (1.3)	1.4 (0.7)	1.6 (0.8)	1.5 (0.7)
Second treatment	1.4 (0.6)	1.0 (0.0)	1.4 (0.6)	2.3 (1.2)	1.5 (0.7)	1.7 (0.9)	1.5 (0.7)
Third treatment	1.4 (0.7)	–	1.8 (1.0)	2.0 (0.0)	1.4 (0.8)	2.0 (1.3)	1.6 (0.8)
Patients completing ≥90% of recommended treatment duration^a^, *n* (%)							
First treatment	84 (94.4)	5 (100)	47 (94.0)	19 (86.4)	184 (93.9)	49 (87.5)	388 (92.8)
Second treatment	37 (94.9)	2 (100)	12 (85.7)	7 (87.5)	81 (94.2)	18 (100)	157 (94.0)
Third treatment	16 (100)	–	4 (100)	4 (100)	33 (100)	7 (87.5)	63 (98.4)

^a^Recommended treatment duration times were 30 min for foot and 60 min for other anatomical locations. For patients treated on the foot, the treatment time corresponding to the percentage duration was 27–33 min for ≥90% to <110% and >33 min for ≥110%. For patients treated on other locations, the treatment time corresponding to the percentage duration was 54–66 min for ≥90% to <110% and >66 min for ≥110%. *CRNP*, cancer-related neuropathic pain; FAS, full analysis set; *HIV-AN*, HIV associated neuropathy; *NPRS*, numeric pain rating scale; *PHN*, postherpetic neuralgia; *NBP*, neuropathic back pain; Other, other non-diabetic PNP; *PONP*, post-operative and post-traumatic neuropathic pain; *SD*, standard deviation

### Pain scores

**Fig. 2:**
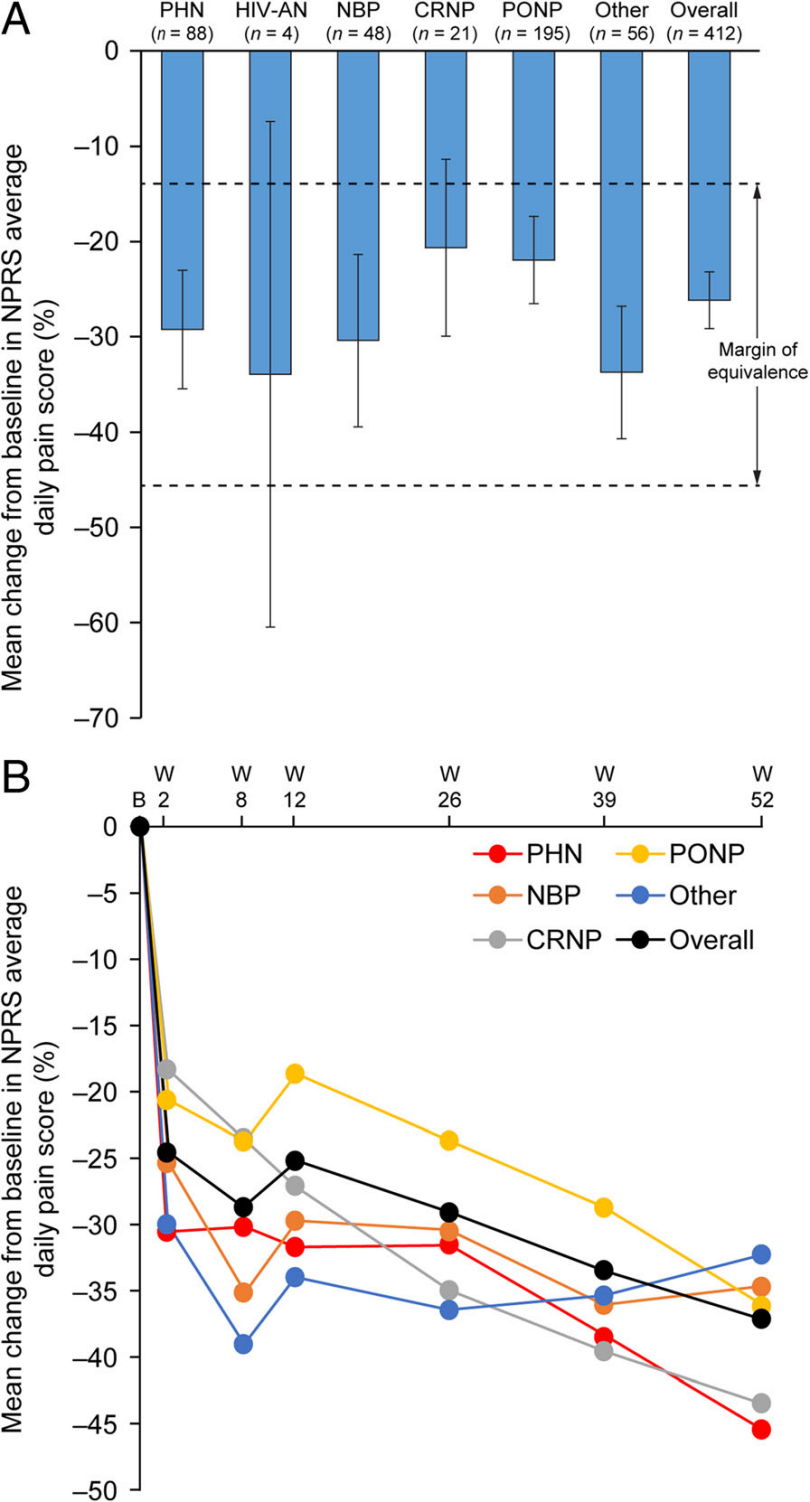
Percentage change in mean NPRS ‘average daily pain’ score following first treatment (**a**) from baseline to Weeks 2 and 8 and (**b**) from baseline to each assessment. The margin of equivalence for percentage change in mean NPRS score was set at ±16% (comparable to a one-point change on the NPRS scale). Error bars represent 95% confidence intervals. CRNP, cancer-related neuropathic pain; HIV-AN, HIV associated neuropathy; NBP, neuropathic back pain; NPRS, numeric pain rating scale; Other, other non-diabetic PNP; PHN, postherpetic neuralgia; PONP, post-operative and post-traumatic neuropathic pain; W, week

Following first treatment with the capsaicin 8% patch, there was an overall 26.6% (95% CI: 23.6, 29.6; *n =* 412) reduction in mean NPRS ‘average pain’ score from baseline to Weeks 2 and 8 (co-primary endpoint) (Table 3; Fig. 2a). The findings of the sensitivity analysis were consistent with this result (–27.7%; *n* = 254). The primary analysis demonstrated equivalence between the PHN group and each of NBP, PONP and ‘other’ groups as the difference did not exceed the pre-defined margin of equivalence (Fig. 2a). Patients who received second and third treatments had similar reductions in their mean NPRS scores of 28.7% (95% CI: 22.9, 34.5; *n =* 161) and 27.3% (95% CI: 18.1, 36.5; *n =* 59), respectively from baseline to Weeks 2 and 12. Overall, patients had a 24.5% (95% CI: 21.1, 27.9; *n =* 401) reduction in their mean NPRS score from baseline to Week 2 and a 37.0% (95% CI: 31.3, 42.7; *n =* 176) reduction to Week 52 (Fig. 2b).

**Table 3:** NPRS ‘average pain’ scores at baseline and Weeks 2 and 8 (FAS)

NPRS ‘average pain’ scores	PHN (*n =* 89)	HIV-AN (*n =* 5)	NBP (*n =* 50)	CRNP (*n =* 22)	PONP (*n =* 198)	Other (*n =* 56)	Overall (*n =* 420)
First treatment							
Baseline, mean (95% CI)	7.1 (6.9, 7.5) *n =* 88	6.4 (5.1, 7.7) *n =* 5	7.3 (6.8, 7.9) *n =* 50	7.2 (6.6, 7.8) *n =* 22	6.8 (6.6, 7.1) *n =* 198	6.9 (6.4, 7.4) *n =* 56	6.9 (6.7, 7.1) *n =* 419
Weeks 2 and 8, mean (95% CI)	4.9 (4.4, 5.4) *n =* 89	4.6 (2.0, 7.3) *n =* 4	4.8 (4.2, 5.5) *n =* 48	5.8 (4.9, 6.7) *n =* 21	5.2 (4.9, 5.5) *n =* 195	4.5 (4.0, 5.1) *n =* 56	5.0 (4.8, 5.2) *n =* 412
Percentage reduction, mean (95% CI)	29.7 (23.4, 36.0)	34.5 (7.6, 61.5)	30.9 (21.7, 40.1)	21.0 (11.6, 30.5)	22.3 (17.7, 27.0)	34.3 (27.3, 41.4)	26.6 (23.6, 29.6)
Second treatment							
Baseline, mean (95% CI)	7.1 (6.5, 7.7) *n =* 39	6.5 (3.6, 9.4) *n =* 2	7.7 (6.7, 8.7) *n =* 15	6.9 (5.9, 7.9) *n =* 8	6.8 (6.4, 7.2) *n =* 97	6.6 (5.7, 7.5) *n =* 19	6.9 (6.6, 7.2) *n =* 180
Weeks 2 and 12, mean (95% CI)	4.7 (4.1, 5.3) *n =* 39	3 (–) *n =* 2	4.3 (2.8, 5.8) *n =* 12	3.4 (1.6, 5.2) *n =* 6	4.9 (4.4, 5.4) *n =* 85	5.1 (4.1, 6.1) *n =* 18	4.8 (4.5, 5.1) *n =* 162
Percentage reduction, mean (95% CI)	30.5 (19.8, 41.2) *n =* 38	51.3 (29.2, 73.4) *n =* 2	44.9 (24.7, 65.1) *n =* 12	51.0 (24.3, 77.7) *n =* 6	25.0 (16.5, 33.5) *n =* 85	21.4 (5.2, 37.6) *n =* 18	28.7 (22.9, 34.5) *n =* 161
Third treatment							
Baseline, mean (95% CI)	6.9 (5.9, 7.9) *n =* 16	–	7.5 (4.6, 10.4) *n =* 4	7.8 (6.3, 9.3) *n =* 4	6.3 (5.8, 6.8) *n =* 38	6.8 (5.6, 8.0) *n =* 8	6.7 (6.3, 7.1) *n =* 70
Weeks 2 and 12, mean (95% CI)	3.9 (2.9, 4.9) *n =* 14	–	5.5 (3.5, 7.5) *n =* 4	5.0 (2.6, 7.4) *n =* 4	4.4 (3.7, 5.1) *n =* 29	5.2 (4.2, 6.2) *n =* 8	4.5 (4.0, 5.0) *n =* 59
Percentage reduction, mean (95% CI)	42.3 (26.8, 57.8) *n =* 14	–	25.4 (15.3, 35.5) *n =* 4	35.5 (8.8, 62.2) *n =* 4	23.1 (9.5, 36.7) *n =* 29	13.6 (–20.8, 48.0) *n =* 8	27.3 (18.1, 36.5) *n =* 59

*CI*, confidence interval; *CRNP*, cancer-related neuropathic pain; *FAS*, full analysis set; *HIV-AN*, HIV associated neuropathy; *NBP*, neuropathic back pain; *NPRS*, numeric pain rating scale; Other, other non-diabetic PNP; *PHN*, postherpetic neuralgia; *PONP*, post-operative and post-traumatic neuropathic pain

A total of 44.4% (*n =* 183) and 26.2% (*n =* 108) of patients were classified as ≥30% and ≥50% responders, respectively, after first treatment (Table 4). Of responders at Week 8, 86.9% (*n =* 159/183) retained responder status at Week 12 after first treatment. There was a small increase in the percentage of responders following re-treatment. The percentage of ≥30% responders after second and third treatment was 49.1% (*n =* 79) and 49.2% (*n =* 29), respectively (Table 4). The percentage of ≥50% responders at second and third treatment was 30.4% (*n =* 49) and 30.5% (*n =* 18), respectively. These findings suggest that the proportion of responders was maintained with each subsequent capsaicin 8% patch treatment.

**Table 4:** Responders after each capsaicin 8% patch treatment

NPRS ‘average pain’ score reduction	Responders, *n* (%)
First treatment (*n =* 412)	
≥30% reduction from baseline to Weeks 2 and 8	183 (44.4)
≥50% reduction from baseline to Weeks 2 and 8	108 (26.2)
Second treatment (*n =* 161)	
≥30% reduction from baseline to Weeks 2 and 12	79 (49.1)
≥50% reduction from baseline to Weeks 2 and 12	49 (30.4)
Third treatment (*n =* 59)	
≥30% reduction from baseline to Weeks 2 and 12	29 (49.2)
≥50% reduction from baseline to Weeks 2 and 12	18 (30.5)

*NPRS* numeric pain rating scale

In a subgroup analysis, patients in the shortest PNP duration quartile of 0–0.72 years had a 36.3% (95% CI: 30.0, 42.6; *n =* 101) reduction in their mean NPRS score from baseline to Weeks 2 and 8 compared with reductions of 23.6% (95% CI: 17.1, 30.1; *n =* 104), 25.0% (95% CI: 19.4, 30.6; *n =* 104), and 21.8% (95% CI: 16.4, 27.2; *n =* 103), in the 0.72–2.1 years, >2.1–5.4 years and >5.4 years quartiles, respectively. Similarly, 62.4% of patients in the shortest PNP duration quartile (0–0.72 years) were ≥30% responders after first treatment, followed by 39.4% of patients in 0.72–2.1 years, 40.4% in >2.1–5.4 years, and 35.9% in >5.4 years PNP duration quartiles, respectively. In patients classified as being in the primary, secondary and tertiary stages of the treatment pathway, the change in mean NPRS scores from baseline to Weeks 2 and 8 was –30.5% (*n =* 80), –28.1% (*n =* 177), and –22.8% (*n =* 155), respectively.

### Time to re-treatment

**Fig. 3:**
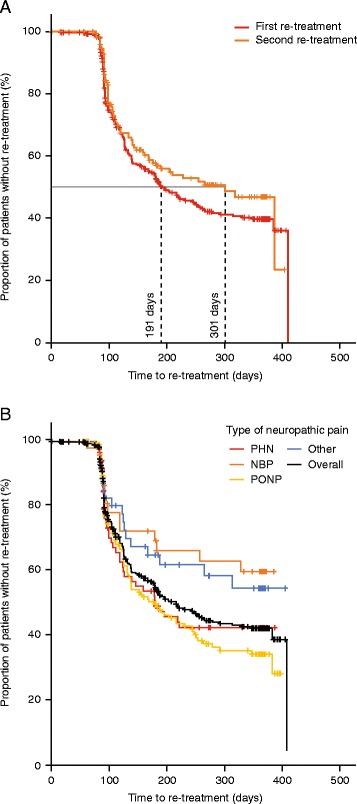
Capsaicin 8% patch re-treatment intervals between (**a**) first and second treatment and second and third treatment; and (**b**) time between first and second treatment by aetiology group. NBP, neuropathic back pain; Other, other non-diabetic PNP; PHN, postherpetic neuralgia; PONP, post-operative and post-traumatic neuropathic pain

Patients had a median time from first to second treatment of 191 days (95% CI: 147, 235; *n =* 181) (co-primary endpoint) and a median time from second to third treatment of 301 days (95% CI: 245, 357; *n =* 70) (Fig. 3a). The median time to second treatment for the PHN and PONP groups was 180 days (95% CI: 116, 244; *n =* 40) and 161 days (95% CI: 120, 202; *n =* 97), respectively (Fig. 3b). Median time to second treatment could not be calculated for NBP and the ‘other’ aetiology groups within the period of the study and the HIV-AN and CRNP aetiology groups were excluded due to low recruitment.

### EQ-5D

The mean EQ-5D health state utility score increased by 0.199 utils at Week 2 (from a baseline score of 0.345 utils), at least two-times greater than the minimally important difference of 0.074 utils [26]. This improvement was maintained to Week 52 following first treatment (Table 5). Responders to capsaicin 8% patch treatment reported the most substantial improvements in HRQoL. At Week 2 after first treatment, ≥30% and ≥50% responders had increases of 0.292 utils and 0.327 utils, respectively in their EQ-5D scores from baseline.

**Table 5:** EQ-5D index scores following first 8% capsaicin patch treatment

EQ-5D index score	Responders at Weeks 2 and 8		Non-responders at Weeks 2 and 8		Overall
	≥30%^a^	≥50%^a^	<30%^a^	<50%^a^	
Baseline, mean (SD)	0.392 (0.352) *n =* 182	0.386 (0.367) *n =* 108	0.306 (0.349) *n =* 229	0.329 (0.347) *n =* 303	0.345 (0.354) *n =* 419
Change from baseline, mean (SD)					
Week 2	0.292 (0.313) *n =* 176	0.337 (0.327) *n =* 104	0.128 (0.297) *n =* 223	0.152 (0.296) *n =* 295	0.199 (0.315) *n =* 400
Week 8	0.323 (0.307) *n =* 165	0.349 (0.321) *n =* 99	0.104 (0.323) *n =* 209	0.147 (0.322) *n =* 275	0.200 (0.333) *n =* 375
Week 12	0.289 (0.352) *n =* 165	0.342 (0.359) *n =* 97	0.111 (0.320) *n =* 196	0.138 (0.325) *n =* 264	0.190 (0.348) *n =* 363
Week 26	0.288 (0.292) *n =* 93	0.309 (0.290) *n =* 62	0.099 (0.339) *n =* 112	0.131 (0.334) *n =* 143	0.185 (0.330) *n =* 206
Week 39	0.306 (0.320) *n =* 78	0.310 (0.321) *n =* 52	0.123 (0.335) *n =* 96	0.161 (0.339) *n =* 122	0.205 (0.340) *n =* 174
Week 52	0.307 (0.327) *n =* 76	0.305 (0.330) *n =* 52	0.114 (0.376) *n =* 100	0.153 (0.373) *n =* 124	0.198 (0.367) *n =* 176

^a^Percentage reduction in average NPRS score. *EQ-5D*, EuroQol 5 Dimension 3-level; *SD*, standard deviation

### PGIC

Analysis of PGIC demonstrated that the majority of patients experienced an improvement (very much, much or slightly) in health status during the study (Table 6). Following first, second and third treatment applications, 61.0% (*n =* 224/367), 74.6% (*n =* 112/150) and 78.7% (*n =* 37/47) of patients, respectively reported improved status at Week 12, while 7.4% (*n =* 27/367), 5.3% (*n =* 8/150) and 4.3% (*n =* 2/47) of patients reported some deterioration. At Week 52, following first, second or third treatment, more than half of all patients had an improvement in PGIC and less than 8% had worsened.

**Table 6:** Patient Global Impression of Change (PGIC) responses

Response	Patients, *n* (%)		
	First treatment	Second treatment	Third treatment
Week 2	*n =* 408		
Improved	258 (63.2)		
No change	117 (28.7)		
Worsened	33 (8.1)		
Week 8	*n =* 368		
Improved	231 (62.8)		
No change	110 (29.9)		
Worsened	27 (7.3)		
Week 12	*n =* 367	*n =* 150	*n =* 47
Improved	224 (61.0)	112 (74.7)	37 (78.7)
No change	116 (31.6)	30 (20.0)	8 (17.0)
Worsened	27 (7.4)	8 (5.3)	2 (4.3)
Week 26	*n =* 207	*n =* 101	*n =* 28
Improved	113 (54.6)	67 (66.3)	23 (82.1)
No change	76 (36.7)	26 (25.7)	3 (10.7)
Worsened	18 (8.7)	8 (7.9)	2 (7.1)
Week 39	*n =* 177	*n =* 81	*n =* 21
Improved	96 (54.2)	50 (61.7)	18 (85.7)
No change	65 (36.7)	24 (29.6)	1 (4.8)
Worsened	16 (9.0)	7 (8.6)	2 (9.5)
Week 52	*n =* 176	*n =* 56	*n =* 7
Improved	93 (52.8)	38 (67.9)	5 (71.4)
No change	71 (40.3)	14 (25.0)	1 (14.3)
Worsened	12 (6.8)	4 (7.1)	1 (14.3)

Improved: slightly, much, very much; Worsened: slightly, much, very much

### Tolerability

Capsaicin 8% patch treatment was generally well tolerated. Over the course of the study, 47 (11.2%) patients reported a total of 91 AEs. The number of patients reporting at least one AE following first, second, third and fourth treatment was 26 (6.2%), 15 (3.6%), 5 (1.2%) and 1 (0.2%), respectively. The most frequently reported AEs were anticipated capsaicin-related application site reactions (8.3%; *n =* 35) including erythema (8.1%; *n =* 34), pain (5.0%; *n =* 21) and pruritus (1.0%; *n =* 4). Twenty-one SAEs were reported in 9 (2.1%) patients; five of these reported for one patient were probably treatment-related (two application site erythema, two application site pruritus and one headache). Four patients died during the study; causes of death were assessed by the treating physicians and were not considered to be treatment-related.

## Discussion

ASCEND was the first real-world study to demonstrate that treatment with the capsaicin 8% patch can provide effective, rapid and sustained pain relief in a heterogeneous population with respect to PNP aetiologies, gender, age, and duration of previous neuropathic pain. A pain response was observed as early as Week 2, in common with previous clinical studies [12, 16, 18]. Long-term follow up of patients enabled the observation that the median time to second treatment was more than 26 weeks and increased to over 43 weeks from second to third treatment. There were also clear benefits in HRQoL and in treating patients with a short history of PNP. In line with other analgesics such as pregabalin and gabapentin [27, 28], observations of the capsaicin 8% patch in routine clinical practice were consistent with findings from clinical trials [12–16].

The mean NPRS ‘average pain’ reduction from baseline to Weeks 2 and 8 (26.6%), and to Weeks 2 and 12 after second and third treatment (28.7% and 27.3%), was consistent with Phase 3 studies of the capsaicin 8% patch in non-diabetic patients with PHN [12, 14] and HIV-AN [13, 16]. These findings are further supported by a randomised, open-label, non-inferiority study of the capsaicin 8% patch versus pregabalin (ELEVATE study) where non-diabetic patients with PHN, peripheral nerve injury or non-diabetic painful peripheral polyneuropathy had non-inferior pain relief versus pregabalin treatment, with a more rapid onset of action, fewer systemic effects and greater patient satisfaction with treatment [18]. In addition, studies performed in parallel to the ASCEND study have reported positive data for capsaicin 8% patch treatment in patients with painful diabetic peripheral neuropathy [29, 30]. A recent European label extension now allows for the use of the capsaicin 8% patch, either alone or in combination with other pain medications, in adults with diabetic PNP. Together, these data confirm that the capsaicin 8% patch provides consistent pain relief in a broad range of PNP aetiologies.

A potential advantage of the capsaicin 8% patch over other treatments is that a single treatment can provide lasting pain relief. In this study, almost half of all patients achieved a clinically important ≥30% reduction [21] in mean NPRS ‘average pain’ by Weeks 2 and 8 following first treatment, which was sustained in patients receiving re-treatment. Significantly, 86.9% of ≥30% responders at Weeks 2 and 8 were also classified as responders at Week 12. These results are concordant with a study in patients with HIV-AN [31], where responders (≥30% reduction in ‘average pain’ from baseline to Weeks 2 and 12) maintained a response for a median time of 17 weeks (95% CI: 13, 27) after a single treatment with the capsaicin 8% patch. Taken together, these data support the conclusion that a clinically important pain response with capsaicin 8% patch treatment is likely to be sustained over time and with successive treatments.

PNP treatment guidelines suggest the use of the capsaicin 8% patch as second-line treatment for localised neuropathic pain [6]. The duration of pre-existing pain and the stage of the treatment pathway, in relation to capsaicin 8% patch treatment, were assessed in this study. Higher levels of pain reduction were observed in patients treated within the primary and secondary stages of the treatment pathway compared with the tertiary stages. Furthermore, the shortest PNP duration quartile (0–0.72 years) had the largest mean percentage reduction in pain intensity from baseline to Weeks 2 and 8, and the highest percentage of responders (≥30% reduction in mean NPRS score). This is consistent with findings from a 12-week non-interventional study (QUEPP), where patients with pre-existing pain of less than 6 months benefited to a greater extent than patients with a longer history of pain [19], indicating that the capsaicin 8% patch may be most effective in earlier stages of PNP treatment or after recent onset of neuropathic pain.

The number of capsaicin 8% patches used per treatment in this study (1.5 patches/treatment) was consistent with the ELEVATE study [18], but considerably lower than that observed in randomised studies (mean 2.3 patches/treatment) [12, 14], despite comparable treatment area sizes. This may be related to differences in the number of patches per pack between the Phase 3 randomised controlled studies (4 patches/pack) and those used in routine clinical practice (1 patch/pack) or the cost of treatment in routine clinical practice versus a clinical trial. The lower number of patches used in this study did not affect the reported efficacy.

Patients with PNP can experience considerable impairment in HRQoL as highlighted by the EQ-5D index at baseline (0.345 utils), which suggests the typical UK adult would choose to forfeit almost two-thirds of their remaining lifespan in order to avoid this state of health [23]. The capsaicin 8% patch improved HRQoL as demonstrated by an improvement in the EQ-5D index and PGIC. The improvement from baseline in the EQ-5D index at Week 2 (0.199 utils) was maintained up to the final measurement at Week 52. It was also observed that responders had the greatest improvements in EQ-5D. Furthermore, 61.0% of patients reported an improved health status at Week 12 as measured by PGIC, similar to results obtained from previous studies in patients with HIV-AN (67%) and PHN (62%) [13, 14]. This result was sustained over successive treatments and up to Week 52 (52.8%).

The capsaicin 8% patch was shown to be well tolerated across a range of aetiologies with over 92% of patients completing at least 90% of the suggested patch application duration at first or subsequent treatments. Similarly, 98% of patients in a previous study with PHN completed at least 90% of the suggested patch application duration [14]. AEs were reported for 11% of patients and most were anticipated application site reactions. The frequency of adverse events reported in other studies of the capsaicin 8% patch was higher (98–99%), but the majority were also application site reactions [12, 14]. Repeated use of the capsaicin 8% patch did not increase the frequency of AEs, supporting findings from an open-label study in patients with HIV-AN who had up to three applications [31].

The strengths of the ASCEND trial include a large and heterogeneous population, a real-world setting, inclusion of patients with different PNP aetiologies and the monitoring of patients for at least one year after treatment and over multiple treatments. A potential limitation of the trial was low patient numbers in the CRNP and HIV-AN groups, which prevented meaningful subgroup analyses. This could have been improved with stratified sampling to increase recruitment in the subgroups. In addition, controlling for longitudinal change in concomitant medication was not possible in this study and could have affected the outcomes reported with the capsaicin 8% patch.

## Conclusions

In conclusion, the use of the capsaicin 8% patch in a real-world, clinical practice setting provided rapid pain relief in patients with various PNP aetiologies. The response to initial treatment and re-treatment was sustained as evidenced by the maintenance of treatment response with re-treatment intervals averaging over 26 weeks. The capsaicin 8% patch was generally well tolerated, usually required less than two patches per treatment, and improved overall HRQoL. Patients in the primary stage of treatment or with short duration of disease had the greatest pain reduction suggesting that patients with PNP may benefit from early treatment with the capsaicin 8% patch. In addition, the capsaicin 8% patch may benefit patients who have inadequate pain relief from systemic therapies or for those suffering intolerable systematic side effects.

## Abbreviations

AE: Adverse event
CI: Confidence interval
CRNP: Cancer-related neuropathic pain
EQ-5D: EuroQol 5 Dimension questionnaire (3-level)
FAS: Full analysis set
HIV-AN: Human immunodeficiency virus-associated neuropathy
HRQoL: Health-related quality of life
NBP: Neuropathic back pain
NeuPSIG: Neuropathic pain special interest group
NIS: Non-interventional study
NP: Neuropathic pain
NPRS: Numeric pain rating scale
PGIC: Patient global impression of change
PHN: Post-herpetic neuralgia
PNP: Peripheral neuropathic pain
PONP: Post-operative and post-traumatic neuropathic pain
w/w: weight for weight
SAE: Serious adverse event
SD: Standard deviation
TRPV1: Transient receptor potential vanilloid type 1
